# Interaction analysis of tight junction proteins by Förster resonance energy transfer is recommended in live cells rather than after paraformaldehyde fixation

**DOI:** 10.3389/fphys.2026.1757655

**Published:** 2026-05-05

**Authors:** Catrin Przibylla-Diop, Marianne Musinszki, Markus Bleich, Susanne Milatz

**Affiliations:** Institute of Physiology, Kiel University, Kiel, Germany

**Keywords:** acceptor photobleaching, claudin-10, false-positive FRET, fixation artifacts, live-cell imaging, membrane protein interactions, paracellular ion transport, paraformaldehyde fixation

## Abstract

The tight junction regulates the paracellular passage of solutes and water. It is primarily constituted by the family of claudins which assemble to huge multimers and basically seal the paracellular cleft. Certain claudins, however, can function as subunits of selective paracellular channels and thereby mediate paracellular ion and water flux across epithelia. In the thick ascending limb of Henle’s loop (TAL), ion handling depends on the spatial separation of paracellular sodium and magnesium transport. Magnesium is conducted via a claudin-16/19 complex, whereas sodium permeation is enabled by claudin-10b which forms autonomous tight junctions without interacting with other TAL claudins. Förster resonance energy transfer (FRET) analysis is a well-established technique to study protein-protein interactions, based on the non-radiative energy transfer between closely positioned donor and acceptor chromophores. FRET has been broadly used to analyze claudin oligomerization as a prerequisite for tight junction formation. Although FRET can be performed in both living and fixed cells, comparability between these conditions remains poorly characterized. While fixed-cell FRET analyses are common for transmembrane ion channels, data for claudins under fixation are lacking. In this study, we evaluated the suitability of paraformaldehyde fixation for analyzing interaction between TAL claudins -10b and -19. Using acceptor photobleaching FRET, we compared the energy transfer efficiencies in live and fixed cells expressing fluorophore-tagged claudins. Fixation markedly altered energy transfer efficiency, leading to false-positive evaluation of apparent claudin interactions. These findings demonstrate that paraformaldehyde fixation compromises the accuracy of FRET measurements and indicate that FRET analyses are best performed in living cells.

## Introduction

### Claudins

Claudins are transmembrane proteins and function as main component of tight junctions (TJ). They were initially described in 1998 (Furuse et al., 1998). To date, 27 human claudin paralogs have been identified (Mineta et al., 2011). A defining feature of claudins is their capacity to assemble into multimeric strands that constitute the TJ. Claudins primarily form a paracellular barrier that seals the space between adjacent cells. Some claudin family members can create size- and charge-selective pores within the TJ strand meshwork. Depending on their specific claudin composition, TJ strands seal against or conduct solutes, ions and water (Furuse et al., 2001, 2002; Van Itallie et al., 2001, 2003, 2006; Amasheh et al., 2002, 2005; Colegio et al., 2002; Nitta et al., 2003; Yu et al., 2003; Angelow et al., 2007; Günzel et al., 2009; Milatz et al., 2010; Rosenthal et al., 2010, 2020; Tamura et al., 2011; Krug et al., 2012; Tanaka et al., 2016).

All claudins exhibit a conserved topology, characterized by four transmembrane helices, two extracellular loops, and intracellular N- and C-termini (Morita et al., 1999). Despite this common structural framework, claudins vary significantly in their tissue expression patterns and their interaction properties (Krause et al., 2008). For example, in the kidney claudin-10b exclusively forms homomers through interactions with other claudin-10b proteins. In contrast, claudin-19 is capable of interacting with multiple claudin partners, including claudin-3 and claudin-16, to form heteromers (Milatz et al., 2017).

Interactions between claudins on adjacent plasma membranes are termed *trans*-interactions, while interactions occurring within the same plasma membrane are known as *cis*-interactions (Piontek et al., 2008). Only the latter can be investigated using Förster resonance energy transfer (FRET), since the maximum effective FRET distance is less than the 10 nm distance between two adjacent cell membranes.

### Förster resonance energy transfer

FRET is a quantum mechanical process first described by Theodor Förster (Förster, 1948). It describes the non-radiative transfer of energy from an excited chromophore, known as the donor, to at least one other chromophore, referred to as the acceptor. The energy transfer occurs when the transition dipoles of donor and acceptor are in resonance, leading to an overlap between the donor’s emission spectrum and the acceptor’s absorption spectrum. In addition to spectral overlap, the spatial distance between the chromophore molecules (e.g., fluorophore-labeled proteins) is essential and determines the probability of energy (i.e. quantum) transfer between donor and acceptor. In 1967, Lubert Stryer and Richard Haugland confirmed the strong dependence of energy transfer on the distance between donor and acceptor, demonstrating that this effect is applicable even on the scale of a few nanometers, thereby establishing its relevance for research in macromolecular biology (Stryer and Haugland, 1967). The key parameter associated with this phenomenon is the Förster radius (*r_0_*), which defines the distance at which 50% of donor excitations are transferred to the acceptor. The efficiency of energy transfer (*E_F_*) is defined using the Förster radius as follows:

(1) $$E_{F}=\;\frac{1}{1+{\left(\frac{r}{r_{0}}\right)}^{6}}$$

where r is the spatial distance between the two fluorophores (Equation 1). In addition to the distance between molecules, the quantum transition also largely depends on the relative orientation of their respective transition dipole, a factor known as the orientation factor (κ^2^). This parameter quantifies the strength of interaction between two transition dipoles based on their relative orientation and can range from 0 to 4. An orientation factor of 4 is achieved when the dipoles are aligned collinearly, indicating the strongest interaction. In cases of (anti-)parallel alignment, κ² equals 1. When the transition dipoles are orthogonal to each other, the orientation factor is 0, signifying no energy transfer despite the proximity of the donor and acceptor.

Moreover, the apparent E_F_ can increase with the number of acceptors within FRET distance of the donor, since higher local acceptor density enhances the likelihood of energy transfer from individual donors. Therefore, the measured E_F_ within the region of interest is a function of the acceptor/donor ratio (Milatz et al., 2015).

Several methods are available to assess the efficiency of energy transfer between fluorophores. The acceptor photobleaching technique requires measuring the donor intensity in both presence and absence of the acceptor fluorophore. The absence of the acceptor is achieved by subjecting it to repetitive irradiation, which induces photobleaching. This process chemically modifies the acceptor molecule, resulting in a loss of its ability to absorb and emit light (Lakowicz, 2006). In this study, cyan fluorescent protein (CFP) and yellow fluorescent protein (YFP) were used as the donor and acceptor (FRET pair), respectively.

### Paraformaldehyde

**Figure 1:**
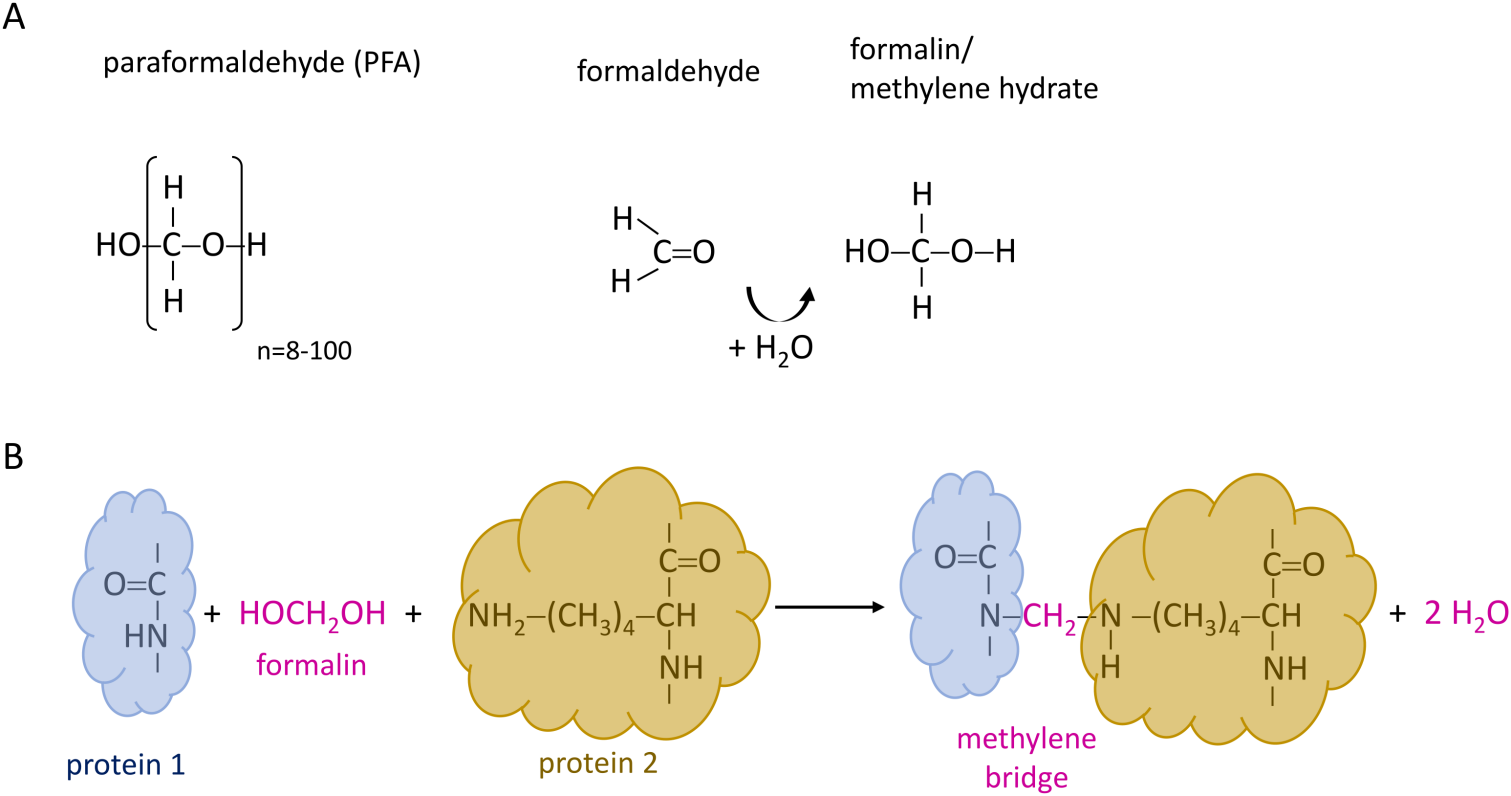
Protein crosslinking induced by paraformaldehyde as described by Kiernan (Kiernan, 2000). **(A)** Chemical structure of paraformaldehyde (PFA), formaldehyde, and formalin. PFA consists of up to ≈100 polymerized formaldehyde monomers. In aqueous solution, formaldehyde is converted to methylene hydrate, referred to as formalin. **(B)** Formalin reacts with amino groups in proteins, including those present in the side chains of arginine, cysteine, histidine, and lysine. Upon release of water, methylene bridges are formed, resulting in covalent crosslinking between proteins.

Paraformaldehyde (PFA) is a common fixative in histological and cytological studies that is used to preserve biological specimens for microscopic analysis. PFA is a polymer consisting of up to 100 formaldehyde monomers (Darvell, 2018). In aqueous solution, formaldehyde is converted to methylene hydrate, also known as formalin. The formalin cross-linking effect depends on formation of methylene bridges between peptides and proteins (**Figure 1**) (Kiernan, 2000). Studies have revealed that these covalent bonds are formed between the amino group of one protein and the amino group of the side-chain of preferably arginine, cysteine, histidine, and lysine, but not between two primary amino groups. Furthermore, the number of cross-links has been shown to increase with time (Kanagy, 1956). Although PFA is one of the most commonly used fixatives to maintain the 3D structure of specimens, PFA-induced cross-linking has a variety of drawbacks, including high toxicity, degradation of nucleic acids, as well as a decrease of the availability of epitopes for antibody-binding (Pabst, 1987; Cox et al., 2006; Moelans et al., 2011), potentially interfering with subsequent immunofluorescence studies.

Although FRET measurements are commonly performed in both living and fixed cells, the comparability of these conditions has not been systematically examined for claudins. Since FRET analyses in PFA-fixed cells offer certain advantages compared to FRET in live cells, we addressed the question whether or not FRET in fixed cells would be a suitable method to study interaction of TAL claudins.

## Methods

### Genetic engineering of fusion proteins

Human claudin-10b (GenBank accession number NM_006984) and human claudin-19 (GenBank accession number NM_148960) coding sequences were inserted into pcDNA3-NECFP-N1 and -NEYFP-N1 vectors (Clontech; GenBank accession number JB978125.1) via *BamHI* and *Eco32I* restriction sites. Expression resulted in claudin proteins, tagged with either ECFP or EYFP at their N termini. N-terminally ECFP- or EYFP-tagged human TREK1 (GenBank accession number NM_001017424) was generated by inserting the coding sequence into a pFAW vector containing 5’ EYFP or ECFP sequences using *SpeI* and *XhoI* restriction sites. Another construct contained an ECFP-EYFP tandem with a linker of 81 base pairs between the open reading frames for both fluorophores. Hereafter, EYFP and ECFP are abbreviated with YFP and CFP, respectively.

### Cultivation, transfection, and fixation of cells

For experiments on fixed cells, HEK-293 (human embryonic kidney) cells were grown on high-precision 1.5H coverslips prior to microscopy. For live cell experiments, cells were seeded on LabTEK imaging chambers with 1.5H glass coverslip bottom (ibidi, Gräfelfing, Germany). In each case, glass was previously coated with poly-L-lysine (Sigma, Saint Louis, Missouri, USA) for better cell attachment. Cells were cultured in Dulbecco’s modified Eagle’s medium (DMEM) supplemented with 10% fetal calf serum (FCS) and penicillin/streptomycin in 5% CO_2_ at 37 °C. One day after seeding, cells had reached sub-confluency and were transiently transfected using polyethylenimine (Polyscience, Hirschberg, Germany). Three hours post-transfection, the mixture was removed and replaced with fresh culture medium. Unless otherwise stated, after twenty-four hours of incubation, cells were either used for live cell-imaging or fixed using 4% paraformaldehyde in PBS with various incubation times (5, 10, 20 minutes), followed by rigorous washing with PBS. Subsequently, coverslips were mounted in Mowiol medium onto slides.

### Imaging

Fixed cells were analyzed one day post fixation using a confocal laser scanning microscope (LSM 880; Carl Zeiss, Germany). For live-cell imaging, cells were transferred into a Hepes-buffered solution [134.6 mM NaCl, 2.4 mM Na_2_HPO_4_, 0.6 mM NaH_2_PO_4_, 5.4 mM KCl, 1.2 mM MgCl_2_, 1.2 mM CaCl_2_, 10 mM Hepes and 10 mM D(+)-glucose, pH 7.4] and maintained at 37 °C. Live-cell imaging was carried out using a confocal laser scanning microscope (LSM 980; Carl Zeiss, Germany). CFP and YFP were excited with argon laser lines at 458 and 514 nm, respectively. Images were acquired using a 63× oil immersion objective (numerical aperture 1.4) with a pixel dwell time of 4.1 µs. Spatial sampling was adjusted according to the Nyquist criterion to ensure optimal resolution. Care was taken to minimize donor bleaching during image acquisition.

Since YFP is excited to a small extent by the 458 nm laser, the detection window for CFP emission was set to 465–490 nm to ensure exclusive detection of CFP. Considering the extended emission spectrum of CFP into longer wavelength ranges, it was not possible to detect pure YFP emission without contamination from CFP. Consequently, a detection window of 525–555 nm was selected, where YFP emission strongly predominates relative to CFP.

### FRET, acceptor photobleaching and data analysis

For all FRET analyses, the acceptor photobleaching method was employed. In case of close proximity between donor and acceptor fluorophores (8 nm at maximum for CFP/YFP), energy transfer enhances the emission of the YFP acceptor while quenching the CFP donor. Destruction of YFP by photobleaching stops the energy transfer and restores the CFP intensity. Photobleaching was achieved by repeated laser scans (iterations) using the 514 nm argon laser line at 100% intensity, applying five pulses for live cells and 100 pulses for fixed cells. Only data from measurements in which acceptor bleaching exceeded 80% were included in the analyses. Before and after acceptor bleaching, CFP and YFP intensities were recorded sequentially in an area of interest to obtain intensity changes due to FRET. For each measurement, two images were captured before and after acceptor bleaching and averaged for the subsequent analysis. FRET efficiency (*E_F_*) was calculated as

(2) $$E_{F}=\frac{\left(I_{A}-I_{B}\right)}{I_{A}}*100$$

where *I_B_* and *I_A_* refer to the CFP intensity before and after the photobleaching, respectively (Equation 2). Considering the dependence of *E_F_* on the acceptor/donor ratio, the fluorescence intensities of a CFP-YFP tandem (ratio 1:1) were measured before and after photobleaching for each experimental series, using the same settings as for the samples. These measurements were used to determine the actual YFP/CFP ratio: The obtained values for YFP emission before bleaching were plotted as a function of CFP values after bleaching. The slope of the linear regression fitted to the individual data points, with an intercept at (0,0), represents the correction factor. To standardize the ratio of YFP to CFP, the measured YFP values of the samples were divided by the obtained correction factor. Subsequently, the *E_F_* values of each potential interaction pair were plotted as a function of their corresponding corrected YFP/CFP ratios (YFP_corr_/CFP).

The E_F_ of claudin/claudin FRET pairs within TJ-like strands increases with the YFP_corr_/CFP ratio and reaches a plateau (Milatz et al., 2015), as a sufficient number of acceptors within the Förster radius increases the probability of energy transfer from an individual donor. To account for this effect, for evaluation and statistical analyses only YFP_corr_/CFP ratios between 0.6 and 3 (plateau range) were included, resulting in E_Fmax_ (Milatz et al., 2017). Statistical analysis was performed using Student’s *t* test with Šidák correction for multiple comparisons and ANOVA.

## Results

### Method evaluation

**Figure 2:**
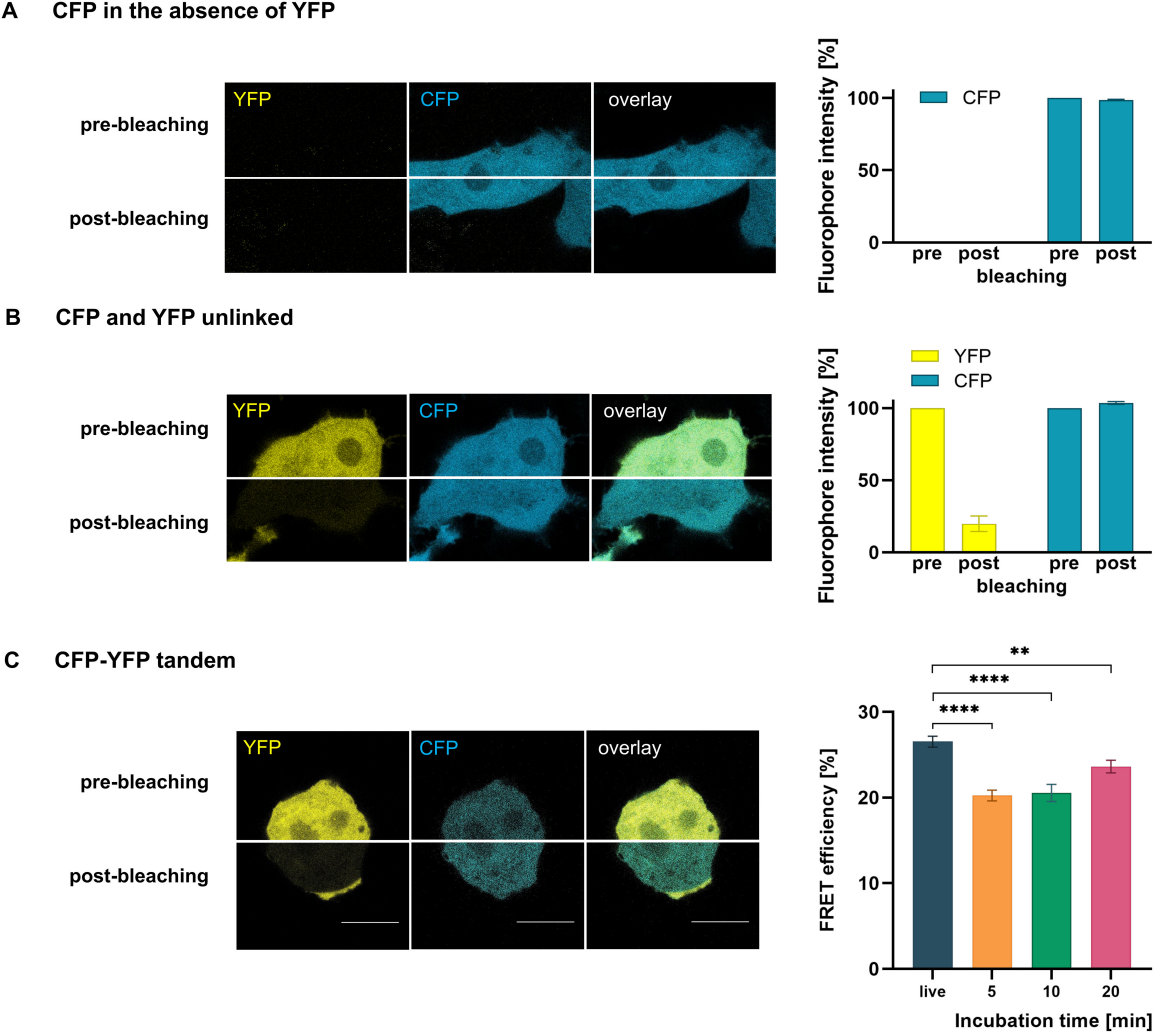
FRET method evaluation in HEK-293 cells. Images illustrate the acceptor photobleaching approach. **(A)** CFP was expressed in the absence of YFP and exposed to the 514 nm laser line used for acceptor photobleaching. Since irradiation did not increase CFP emission (bar graph), the bleaching protocol had no detectable effect on the CFP fluorophore. **(B)** Photobleaching of unlinked CFP and YFP proteins did not result in substantial CFP emission (bar graph), excluding artifactual signals due to crowding or dimerization effects. **(C)** A CFP–YFP tandem construct was subjected to photobleaching, resulting in robust FRET. The tandem with its defined 1:1 stoichiometry, served as a normalization reference to determine the actual YFP/CFP ratio (YFP_corr_/CFP) for the evaluation of membrane proteins. The normalized values were used for all subsequent analyses. Fixation decreased the FRET efficiency E_F_ of the tandem (bar graph, n=28-66). Scale-bar: 10 µm. Bar graphs show mean ± SEM. **P < 0.01; ****P < 0.0001.

For FRET measurements in fixed cells, several systematic errors and artifact sources unrelated to the protein of interest have been reported. We therefore first assessed their potential impact in our experimental setup. Specifically, we examined whether irradiation with the 514 nm laser line used for acceptor photobleaching enhances CFP emission, as previously described (Rodighiero et al., 2008). To that end, CFP was expressed in the absence of YFP and subjected to photobleaching at 514 nm under standard FRET conditions (**Figure 2A**). As a result, CFP fluorescence intensity remained unchanged after irradiation (E_F_=-1.3 ± 0.5%; n=5), indicating that the photobleaching protocol did not alter the CFP fluorophore. Neither did we observe photoconversion of YFP into a CFP-like species upon 514 nm irradiation, as no signal was detected in the CFP channel when YFP was expressed alone.

To further exclude artifactual FRET, we evaluated the potential for FRET between CFP and YFP when both fluorophores were co-expressed as individual, unlinked proteins in HEK-293 cells (**Figure 2B**). We did not measure E_F_ values exceeding 5% (E_F_=3.3 ± 0.9%; n=6), meaning that no substantial energy transfer occurred between unlinked CFP and YFP.

A further set of experiments was conducted to determine the number of bleaching cycles required to achieve an acceptor bleaching efficiency of ≥80%. In live cells, five irradiation cycles were sufficient to meet this threshold. However, in fixed conditions, 100 cycles were necessary to reduce YFP intensity by at least 80%, indicating that photobleaching is significantly more efficient in live cells compared to fixed cells.

Gustavson demonstrated that PFA-induced crosslinks increase over time (Kanagy, 1956). In order to investigate whether fixation time affects E_F_, we performed FRET measurements on the CFP-YFP tandem protein after fixation with varying durations of 5, 10, and 20 minutes. The tandem protein was localized within the cytoplasm and showed clearly positive E_F_ values in live and in fixed cells (**Figure 2C**). However, fixation resulted in a significant decrease in E_F_ compared to live cell conditions (E_F_=26.53 ± 0.63%), irrespective of the fixation duration (E_F_5’_=20.24 ± 0.62%, E_F_10’_=20.54 ± 1.01%, E_F_20’_=23.63 ± 0.74%).

### FRET measurements of membrane proteins in live versus fixed HEK-293 cells

**Figure 3:**
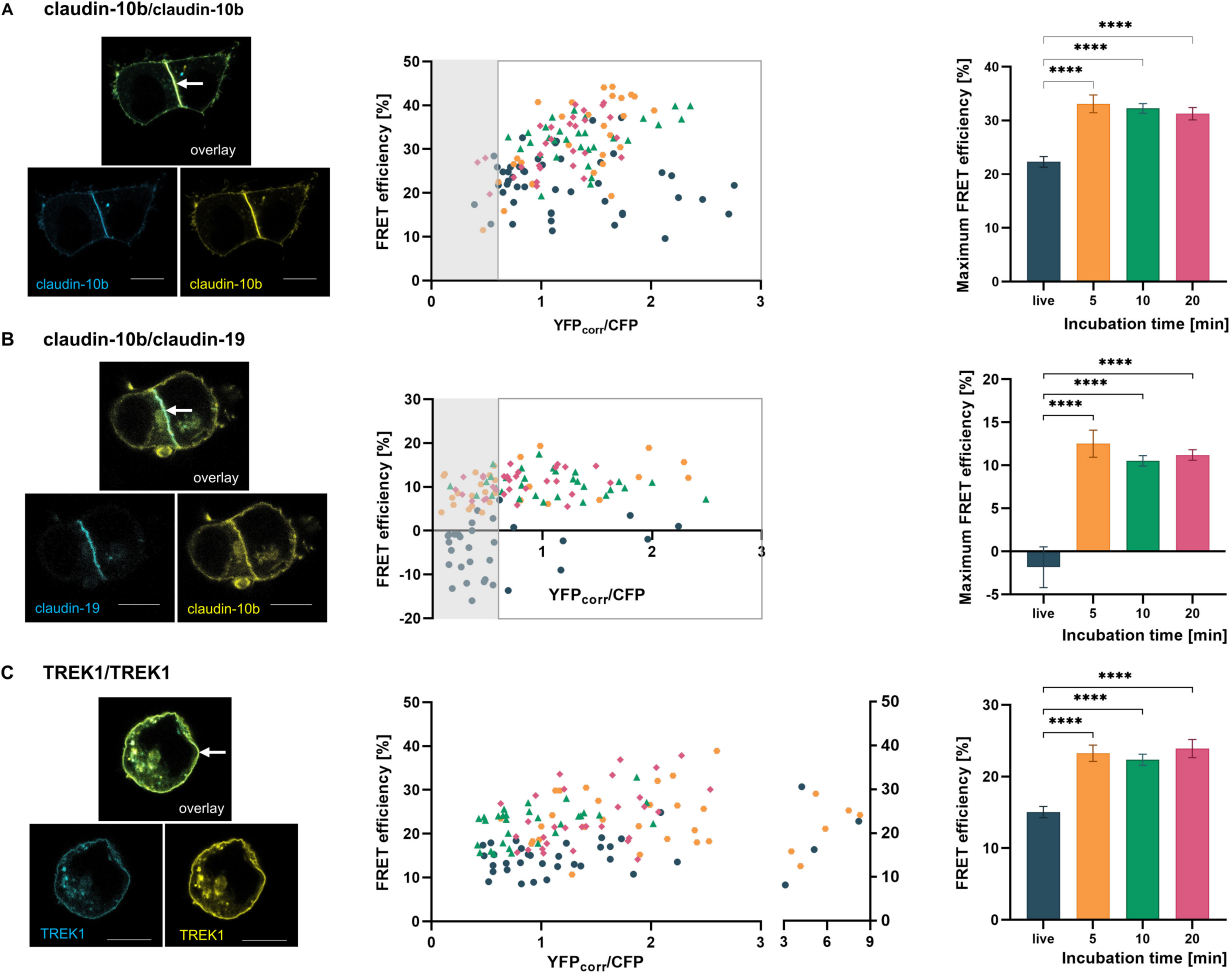
FRET analysis of membrane protein-protein interactions in live and fixed cells. Fusion proteins were co-expressed and analyzed either in live cells or following fixation with PFA for 5, 10, or 20 minutes. **(A)** CFP-claudin-10b/YFP-claudin10b interaction was measured at cell-cell-contacts where TJ-like strands are formed (arrow). Since E_F_ depends on the acceptor/donor ratio, it was plotted as a function of YFP_corr_/CFP. E_F_ values within the plateau range (YFP_corr_/CFP between 0.6 and 3) were averaged and defined as E_Fmax_ (shown in the bar graph; n=26-44). Fixation increased the E_Fmax_ compared to live cell measurements. **(B)** CFP-claudin19/YFP-claudin-10b localize to the same cell-cell-contact in HEK 293 cells (arrow) but are not able to interact. In fixed cells they show false-positive E_Fmax_ values (bar graph, n=8-23). **(C)** CFP-TREK1/YFP-TREK1 was analyzed within the cell membrane (arrow). Please note the adjusted scaling applied to high YFP_corr_/CFP ratios. All E_F_ values were included in the evaluation (n=30-35). Fixation resulted in a significant increase in E_F_. In all approaches, the duration of PFA exposure showed no eminent impact on E_F_. Scale-bar: 10 µm. Bar graphs show mean ± SEM. **P < 0.01; ****P < 0.0001.

FRET interaction analyses of claudins were carried out at cell-cell-contacts where heterologous claudins can form TJ-like strands. As a positive control for cis-interacting claudins, we selected the combination CFP-claudin-10b/YFP-claudin-10b and compared the E_Fmax_ values obtained in fixed cells with those measured under live-cell conditions (**Figure 3A**). Whereas in living cells *E_Fmax_* of claudin-10b/claudin-10b was similar to previously published values (*E_Fmax_* = 22.3 ± 0.97%) (Milatz et al., 2017), fixation led to markedly increased *E_Fmax_* values, independent of PFA incubation time (*E_Fmax_5’_* = 33.1 ± 1.64%, *E_Fmax_10’_* = 32.25 ± 0.9%, *E_Fmax_20’_* = 31.28 ± 1.15%).

The combination of claudin-10 and claudin-19 has been shown to be incapable of *cis*-interaction (Milatz et al., 2017). Therefore, it was used as negative control. As expected, our live-cell measurements confirmed the absence of FRET (*E_Fmax_* = -1.83 ± 2.36%) upon co-expression (**Figure 3B**). In contrast, fixation of claudin-10/claudin-19 expressing cells led to a marked increase in CFP emission after photo bleaching, yielding E_Fmax_ values of *E_Fmax_5’_* = 12.52 ± 1.58%, *E_Fmax_10’_* = 10.54 ± 0.6%, and *E_Fmax_20’_* = 11.21 ± 0.61%. Thus, despite their known inability to interact, claudin-10b/claudin-19 showed E_Fmax_ values usually interpreted as cis-compatibility, with a broad range of values, spanning from 5.5% to 19.34%.

To elucidate whether differences between living and fixed cells are also present in a non-TJ membrane protein, we tested the transmembrane potassium channel TREK1 which forms homodimers (Fink et al., 1996; Lolicato et al., 2017). FRET analysis of TREK1/TREK1 within the plasma membrane of living cells revealed a clear interaction (*E_F_* = 15.05 ± 0.79%) indicating dimerization (**Figure 3C**). However, a notable enhancement in E_F_ was observed in fixed cells which was significant across all fixation durations examined (*E_F_5’_* = 23.26 ± 1.14%, *E_F_10’_* = 22.34 ± 0.78%, *E_F_20’_* = 23.9 ± 1.26%).

## Discussion

The particular compatibility of different claudins determines their assembly within TJ strands and thereby controls the permeability properties of the TJ. In the TAL, ion handling is based on the spatial separation of claudin-10b and claudin-16/19 TJs, responsible for paracellular sodium or magnesium transport, respectively. Sodium conductance is enabled by claudin-10b which forms autonomous tight junctions and is unable to physically interact with other TAL claudins. Magnesium reabsorption on the other hand is mediated by the TJ complex of claudin-16 and -19, spatially separated from claudin-10b-formed TJs (Milatz et al., 2017). In contrast to the conditions in native TAL cells, claudin-10b and claudin-19 localize to the same cell-cell contact when heterologously expressed in HEK-293 cells, which allows FRET measurements there (**Figure 3B**). Still, they are not capable to cis-interact or form joint TJ-like strands.

The acceptor photobleaching approach is widely used to investigate claudin oligomerization as a prerequisite for TJ formation. To ensure methodological robustness, we systematically assessed potential sources of artifacts. Previous reports described altered CFP emission after photobleaching in fixed, mounted, and nail polish–sealed samples, partially attributed to the sealant solvent, but still detectable in unsealed preparations (Rodighiero et al., 2008). In contrast, we observed no increase in CFP emission after irradiation in Mowiol-mounted, unsealed samples, nor in live cells, indicating that under our conditions the CFP fluorophore was not affected by the bleaching protocol. We also did not detect photoconversion of YFP into CFP-like species, as reported previously (Valentin et al., 2005; Raarup et al., 2009), consistent with other studies (Thaler et al., 2006; Rodighiero et al., 2008).

To further exclude artifactual FRET, we analyzed CFP and YFP co-expressed as unlinked cytoplasmic proteins in HEK-293 cells (**Figure 2B**). Molecular crowding may increase fluorophore proximity and can even promote dimerization, as previously reported for green fluorescent protein (Tsien, 1998). Similar spontaneous dimer formation may also occur with the GFP derivatives CFP and YFP and could be further stabilized by fixation. Contrary to previous reports (Rodighiero et al., 2008), but in agreement with others (Anikovsky et al., 2008), E_F_ values remained below 5% in both live and fixed cells. These results argue against artifactual FRET arising from crowding- or dimerization-related effects in our system. Importantly, even at sites of claudin membrane enrichment, no FRET was detected between the non-compatible claudins -10 and -19 under live-cell conditions (**Figure 3B**). Taken together, the positive E_F_ values measured for membrane proteins, which clearly exceeded the 5% threshold in live cells, reflect genuine cis-interactions rather than nonspecific proximity effects.

PFA fixation before performing FRET measurements is commonly used when examining protein-protein interactions. Main advantages of fixation are restricted diffusion and absent movement of the cell, potentially leading to a more precise analysis of the region of interest. Given the substantial scatter in obtained E_F_ values, this would be a worthwhile objective. On the other hand, in our experiments, data scatter was not considerably reduced under fixed conditions.

One significant experimental drawback we observed in fixed cells was the high number of irradiation cycles required for a solid bleaching effect. Acceptor bleaching was much more efficient in living state than in fixed conditions, as the bleaching process is strongly influenced by the presence of molecular oxygen in the environment (Kasche and Lindqvist, 1964).

Nevertheless, the most important problem of fixation lies in the potential alteration of cell morphology, including the shape and volume of the cell, which may result in inaccurate data. Notably, E_F_ was enhanced in all fixed membrane protein samples tested (**Figure 3**). When expressing claudin-10b and claudin-19 simultaneously in HEK-293 cells, we obtained no substantial energy transfer at cell-cell contacts of living cells, which is in good agreement with former data that show a missing capability for this combination to interact in *cis* (Milatz et al., 2017). However, PFA-induced fixation of the cells resulted in a marked energy transfer, revealing a false-positive detection of *cis*-interactions between claudin-10b and claudin-19 (**Figure 3B**).

The increased E_F_ observed after fixation suggests either a change in the orientation of the fluorophore transition dipoles or, more likely, a reduced distance between the FRET pair. However, based on the current data, these two effects cannot be experimentally distinguished. Because of the steep distance dependence of FRET (Equation 1), even minor reductions in intermolecular spacing can substantially increase E_F_. Given the markedly higher osmolality of a 4% PFA solution (1630 mosmol/kg) compared to that of the cytoplasm and the physiological cellular environment (290 mosmol/kg), one possible mechanism is a transient cell shrinkage upon exposure to PFA. Concurrently, crosslinking is initiated, leading to the stabilization of this temporarily shrunken state. Previous studies with L929 cells have demonstrated that PFA-treated cells exhibited a slight reduction in size compared to live cells, accompanied by a decreased cell volume and adhesion area. Additionally, the surface roughness of PFA-treated cells was increased, which has been attributed to the crosslinking effect and potential aggregation of plasma membrane proteins (Kim et al., 2017). The high concentration of claudins in the plasma membrane suggests that even minimal cellular shrinkage could result in increased proximity of these proteins.

A potential contribution of fixation-induced changes in dipole orientation should also be considered. The relative alignment of donor and acceptor transition dipoles is captured by the orientation factor κ^2^, which directly affects the Förster radius r_0_ and, consequently, E_F_ (Equation 1). As r_0_ also depends on the donor quantum yield (QY), the refractive index of the medium (n), and the spectral overlap between donor emission and acceptor absorption (J), alterations in the local physicochemical environment upon fixation could potentially affect it (Lakowicz, 2006). However, since all parameters determining r_0_ enter with a sixth-root dependence (r_0_^6^​∼QY​·κ^2^·J·n^−4^), such effects are likely smaller than distance-dependent changes. Nevertheless, they may still contribute modestly to the observed increase in E_F_.

**Figure 4:**
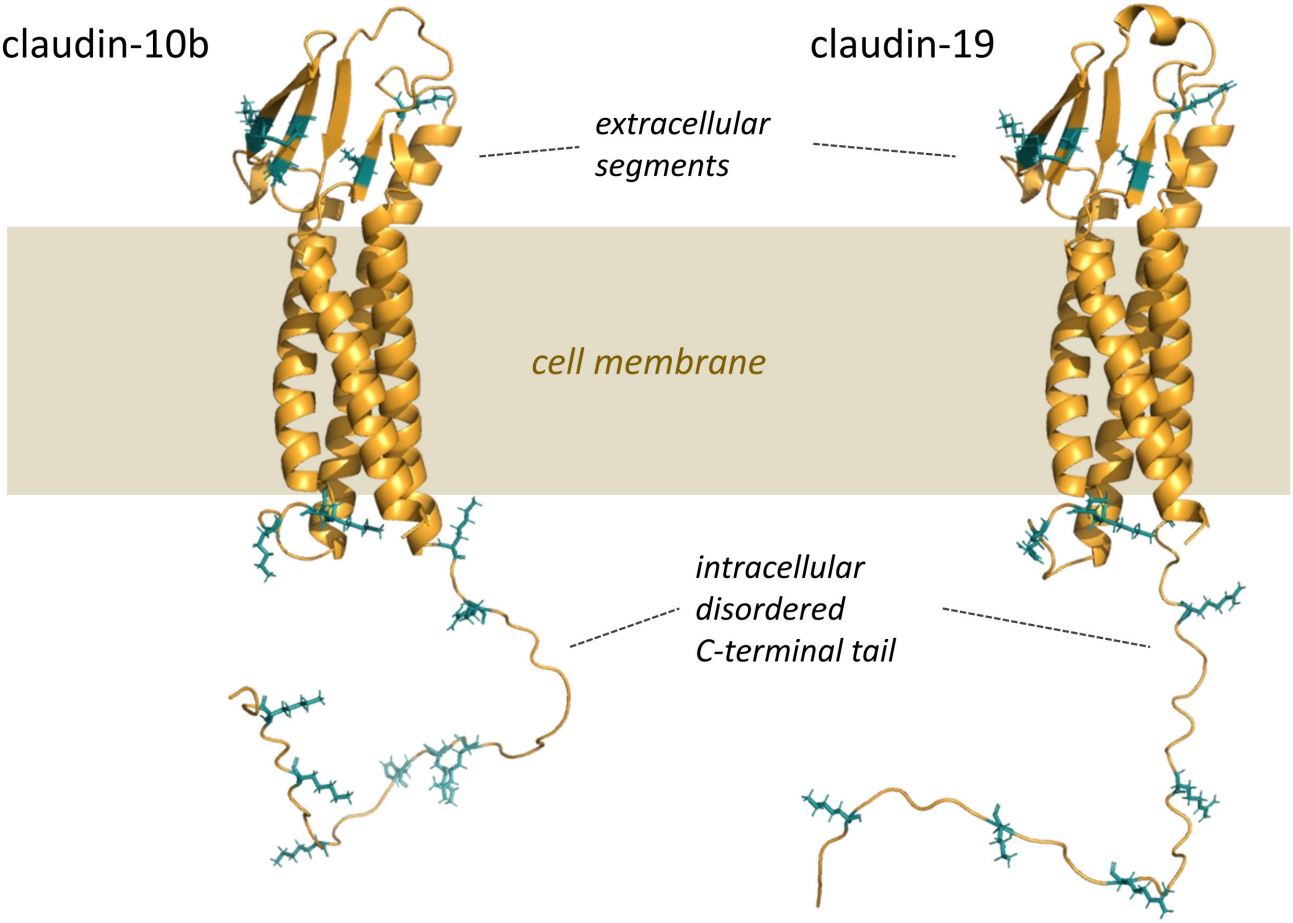
Side view of a model of claudin-10b and claudin-19 highlighting putative formalin crosslinking sites. Formalin forms covalent bonds between the amino groups of different proteins, with a preference for the side chains of arginine, cysteine, histidine, and lysine, which are highlighted in blue.

Moreover, PFA fixation may even promote the formation of claudin aggregates. Crosslinking could occur both, intra- and extracellularly, as claudins contain segments located on both sides of the plasma membrane. In general, claudins present multiple potential crosslinking sites that are preferentially targeted by formalin and that in turn may lead to an effective fixation (**Figure 4**). As a result, the distance between donor and acceptor could further decrease, leading to a higher probability of FRET. The same consideration applies to TREK1 ion channels, which are also anchored to the plasma membrane. For claudins which potentially form multimers within TJ-like strands and have more than one potential interaction partner in close proximity the effect of fixation artifacts on E_F_ might be even more pronounced.

In contrast to claudins and TREK1, the E_F_ of the CFP-YFP tandem protein was consistently lower in fixed cells compared to live cells (**Figure 2C**). The CFP-YFP tandem protein is localized intracellularly and not embedded in the plasma membrane. Consequently, the tandem protein with a linkage of 27 residues between CFP and YFP, possesses all degrees of freedom and can theoretically diffuse freely within the cytoplasm. Thus, CFP and YFP may not necessarily come into closer proximity in response to cell shrinkage. Moreover, fixation could potentially influence fluorophore orientation or restrict the conformational freedom of the linker, thereby anchoring the tandem fluorophores in an unfavorable position and reducing the efficiency of energy transfer. This finding is consistent with previous studies by Anikovsky *et* al (Anikovsky et al., 2008), but contradicts the results of Rodighiero *et* al (Rodighiero et al., 2008), who reported no significant decrease in E_F_ in fixed and mounted cells compared to live cell conditions. Differences between studies may be explained by variations in fixation and permeabilization procedures, mounting media, and other experimental parameters. Future experiments comparing tandem constructs with rigid and flexible linkers would provide a direct test of whether fixation affects FRET by restricting linker mobility.

Our results raise the question how to overcome the problems of fixation in FRET analyses. It is rather questionable whether a reduction in PFA concentration with incomplete fixation would help to avoid cell shrinkage and misrepresentation of membrane protein distances. Given that fixation of whole cells begins at PFA concentrations of ≈0.1% and is complete at ≈4% (Kim et al., 2017), lower PFA concentrations may lead to incomplete crosslinking and thereby compromise the benefits of fixation.

The observation of higher apparent FRET efficiencies in fixed samples could be corrected by comparison to stringent positive and negative controls to identify a suitable threshold for considering a certain E_Fmax_ value as positive interaction. Subtracting the E_Fmax_ of the claudin-10b/claudin-19 negative control as baseline, the E_Fmax_ for the claudin-10b/claudin-10b homomer would be ≈20%, approximately the magnitude for this combination in live cells. However, this correction does not overcome the false positive result in fixed cells when no previous data are available for comparison.

Notably, as fixation can alter protein conformation, spatial organization, fluorophore orientation, or fluorescence lifetime and thereby influence E_F_, it is conceivable that similar fixation-related artifacts are not limited to acceptor photobleaching but could also affect fluorescence lifetime-based FRET approaches such as FLIM. Although the magnitude of the effect may vary depending on the fluorophore pair and cellular context, fixation-induced changes in FRET are likely not restricted to the specific system examined here. Measuring FRET in live cell experiments appears to be the most reliable and authentic approach and should be preferred.

In conclusion, we demonstrate that PFA fixation of cells affects intermolecular FRET at the plasma membrane as well as intramolecular FRET in the cytoplasm. We therefore recommend performing FRET-based interaction analyses in living cells whenever possible. If fixation is required, stringent controls and careful threshold definition are essential to avoid misinterpretation.

## Data Availability

The data supporting the findings of this study are available in the Zenodo repository at https://doi.org/10.5281/zenodo.19705185. Additional data can be made available upon reasonable request from the corresponding author.
